# A Comprehensive Review of Pathophysiological Link Between Non-alcoholic Fatty Liver Disease, Insulin Resistance, and Metabolic Syndrome

**DOI:** 10.7759/cureus.75677

**Published:** 2024-12-13

**Authors:** Eudith Januario, Aly Barakat, Abhivanditha Rajsundar, Zahra Fatima, Varda Nanda Palienkar, Arjun V Bullapur, Sunchandandeep Singh Brar, Punam Kharel, Mishal Mohammed Koyappathodi Machingal, Amena Backosh

**Affiliations:** 1 Medicine, American University of Antigua, Osbourn, ATG; 2 Internal Medicine, Medway NHS Foundation Trust, Gillingham, GBR; 3 Medicine, Tbilisi State Medical University, Tibilsi, GEO; 4 Medicine, Dr. VRK Women's Medical College, Aziznagar, IND; 5 Medicine, Mahsa University, Selangor, MYS; 6 Medicine, MS Ramaiah Medical College, Bengaluru, IND; 7 Medicine, Department of Health and Family Welfare Punjab, Chandigarh, IND; 8 Medicine, Sir Salimullah Medical College, Dhaka, BGD; 9 Medical College, Government Medical College Kozhikode, Kozhikode, IND; 10 Orthopedics, Medway Maritime Hospital, Gillingham, GBR

**Keywords:** diabetes and metabolic syndrome, diabetes type 2, insulin resistance and metabolic syndrome, nafld nash statins nash and statins nash, non-alcoholic fatty liver disease

## Abstract

Non-alcoholic fatty liver disease (NAFLD) is a chronic condition characterized by hepatic steatosis in the absence of significant alcohol consumption and is increasingly recognized as the hepatic manifestation of metabolic syndrome (MetS). This review aims to explore the molecular mechanisms underlying the interaction between NAFLD, insulin resistance (IR), and MetS, with a focus on identifying therapeutic targets. A comprehensive review of existing literature on NAFLD, IR, and MetS was conducted. The review indicates that IR contributes to hepatic lipid accumulation through increased lipolysis, elevated free fatty acid flux, and impaired fatty acid oxidation, while MetS exacerbates the condition by promoting visceral adiposity, chronic inflammation, and impaired lipid metabolism. Additionally, dysbiosis and increased intestinal permeability in the gut-liver axis worsen IR, leading to a vicious cycle of metabolic dysfunction. In conclusion, addressing these interconnected pathways could enhance therapeutic strategies and reduce the burden of NAFLD-related complications.

## Introduction and background

Non-alcoholic fatty liver disease (NAFLD) can be distinguished by the variety of histological changes, particularly lipogenesis along with hepatic steatosis, where there is no excessive alcohol intake. It is prevalent in 14% - 30% of the general population, and it occurs across all ethnicities and age groups [1]. 

A chronic liver disease, most widespread in Western nations, is non-alcoholic fatty liver disease (NAFLD), which has been increasing in prevalence and incidence over time, placing a heavy clinical and financial burden on healthcare systems [2]. In a great number of patients, NAFLD correlates with various metabolic comorbidities, such as dyslipidemia, type 2 diabetes mellitus (T2DM), or obesity, which is often viewed as the metabolic syndrome’s hepatic clinical sign [2].

The co-occurrence of insulin resistance, atherogenic dyslipidemia (low high-density lipoprotein cholesterol, high triglyceride), obesity, and hypertension is known as metabolic syndrome [3]. NAFLD is regarded as a multisystem illness as it is impacted by both hereditary and environmental factors. Among the obesogenic society, over 25% of the population globally have NAFLD, a prevalence that is steadily rising [4,5].

Apart from the known role of insulin resistance and obesity, genome-wide studies demonstrated a substantial correlation between various forms of genes within patatin-like phospholipase domain-containing protein 3 (PNPLA3) rs738409 along with transmembrane 6 superfamily 2 human gene (TM6SF2) rs 58542926 with liver fat [5]. 

NAFLD has the potential to progress to cirrhosis and other deadly cardiovascular complications. Currently, there are no FDA-approved medicines to treat NAFLD. The goal of this review article is to scrutinize the interaction between insulin resistance and NAFLD along with its implications in managing the Metabolic syndrome. 

## Review

Pathophysiology of NAFLD

Insulin resistance is a critical pathogenic element in a variety of metabolic diseases, such as type 2 diabetes mellitus, distinguished by diminished sensitivity of insulin-sensitive tissues to normal insulin levels physiologically. The fundamental mechanism of insulin resistance is still incomprehensible, though many plausible theoretical postulations have been put forth. This article describes the suggested pathways that explain insulin resistance, particularly ectopic accumulation of lipids in the liver and skeletal muscle, and inflammation along endoplasmic reticulum stress [6].

Type 2 diabetes mellitus (T2DM) is primarily defined as a non-physiological increase of plasma glucose levels, which is preceded by insulin resistance. Persistent hyperinsulinemia in the prediabetic stage leads to destruction of β-cells, which ultimately results in T2DM [7]. Insulin-resistant tissues lack the glucose-regulating properties of insulin at normal plasma levels, that is, inhibition of hepatic glucose production (HPG), plasma glucose uptake at a cellular level, lipolysis, and net glycogen synthesis [8]. The liver and adipose tissues are crucial for glucose-induced insulin signaling, whereas skeletal muscle is a quantifiably necessary organ in disposing of insulin-mediated glucose; thus, these tissues are vital in understanding the pathways behind insulin resistance [6].

The principal risk factor in T2DM is obesity as determined by various epidemiological studies, having an 80-fold increased risk in obese people to develop the illness [6]. Furthermore, in individuals who are BMI- and age-matched, it has been demonstrated that insulin sensitivity and plasma fatty acid content are inversely related [9]. Insulin resistance and T2DM are majorly caused by excessive lipid buildup brought on by obesity, according to this and numerous other studies. The process by which insulin resistance advances as a result of an abundance of lipids has been explained extensively.

The Randle cycle (glucose-fatty acid cycle) 

As Randle et al. [10] postulated in 1963, insulin resistance induced by lipids occurs in skeletal muscle, described as the impaired glucose uptake that is caused by restricted utilization of insulin-stimulated glucose due to elevated fatty acid oxidation. This is also called the Randle cycle or glucose-fatty acid cycle. It has been determined that the upsurge of fatty acid oxidation in obesity hinders the usage of glucose as the fuel product by preventing vital glycolytic enzymes from functioning. Randle et al. [10] also determined that the oxidation of fatty acid might raise the amounts of mitochondrial acetyl-coenzym A (CoA) levels via β-oxidation, which would halt the pyruvate dehydrogenase. This process potentially inhibits phosphofructokinase (a critical glycolytic enzyme), raises intracellular citrate levels, and results in the accumulated intramyocellular glucose-6-phosphate. Consequently, this accumulation hinders hexokinase activity, causing a rise in intramyocellular glucose along with a decrease in glucose uptake. Additionally, studies have repeatedly shown that in the rat heart and diaphragm muscles, fatty acid infusions increase intramyocellular glucose-6-phosphate concentrations while decreasing the utilization of myocellular glucose [11-13].

Based on the glucose-fatty acid cycle, fatty acids should increase levels of glucose-6-phosphate and glycogen production while preventing glycolysis. In the muscles of healthy individuals, subjection to raised plasma levels of fatty acid led to resistance to insulin along with lower levels of glucose-6-phosphate [14]. Furthermore, individuals with T2DM dealing with chronic insulin resistance have been found to have reduced oxidation of glucose and insulin-stimulated muscle glycogen synthesis [15,16]. The findings indicate that not all the elements of insulin resistance deficits are fully explained by the glucose-fatty acid cycle.

Hexosamine biosynthesis pathway

Despite evidence that muscular acetyl-CoA and citrate levels increase with enhanced fat oxidation, the glucose-fatty acid cycle is insufficient to elaborate insulin resistance induced by lipids. This implies a different mechanism modulation that transports the glucose regardless of the levels of glucose-6-phosphate in the muscular system.

Insulin resistance can also be explained by the hexosamine biosynthesis pathway (HBP). Primarily generated by glucose-6-phosphate, fructose-6-phosphate undergoes preferential conversion to fructose-1,6-bisphosphate during glycolysis. However, glutamine:fructose-6-phosphate amidotransferase (GFAT), which is a rate-limiting enzyme in HBP, converts about 5% of fructose-6-phosphate into glucosamine-6-phosphate [17]. Uridine 5'-diphosphate N-acetylglucosamine (UDP-GlcNAc) is made from glucosamine-6-phosphate, which serves as a donor sugar nucleotide for the glycosylation and O-GlcNAcylation of proteins as well as lipids [17]. By altering enzyme activity or gene expression, these changes, in particular, O-GlcNAcylation, may have an impact on specific proteins [18].

Additionally, levels of UDP-GlcNAc are known to be increased in skeletal muscle by hyperglycemia and hyperinsulinemia [19,20]. Moreover, it has been discovered that the pancreas, skeletal muscles and corneas of rats with diabetes or insulin resistance had elevated O-GlcNAc levels [21,22]. Mice with a decrease in glucose clearance showed that whole-body insulin resistance was caused by overexpression of GFAT in skeletal muscle or adipose tissue [23].

The highly credible pathway connecting HBP with insulin resistance is O-GlcNAcylation, whereas the molecular events of HBP in insulin resistance continue to be ill-defined. Protein activity and signaling transduction may be regulated by O-GlcNAcylation within proteins, which might clash with phosphorylation for binding sites [24]. O-GlcNAc alters the elements of the signaling cascade of insulin such as IRS-1/2, PI3K, PDK1, and Akt [25,26]. Moreover, O-GlcNAc is altered in the course of insulin resistance induced by glucosamine, identified as mammalian uncoordinated-18c (Munc18-c), a necessary protein aiding in the translocation of insulin-stimulated GLUT4. Additionally, it has been discovered that diabetes causes O-GlcNAcylation of FOXO1, a crucial transcriptional component for gluconeogenic genes inside the liver [27,28].

Ectopic lipid accumulation

Obesity is among the known precursors for T2DM; however, several pieces of research suggest that accumulation of lipid ectopically within peripheral tissues, especially the skeletal muscle and liver, can exacerbate insulin resistance, with the inadequacy of visceral adiposity [29]. Non-alcoholic fatty liver disease (NAFLD) is reported to be present in more than 70% of obese people having T2DM and nearly all patients with NAFLD exhibit both hepatic insulin resistance and T2DM [30]. Furthermore, hepatic insulin resistance is effectively reversed in NAFLD, T2DM, and lipodystrophy by therapies that lower intrahepatic triglyceride levels by leptin therapy or mild weight loss [31]. Insulin resistance can be better predicted by intramyocellular and intrahepatic lipid levels than by the volume of visceral adipose tissue. This implies that higher lipid buildup in the skeletal muscle and liver may cause disruption with insulin signaling that results in insulin resistance [32,33].

Multiple investigations on animals demonstrate that the ectopic accumulation of lipids in the skeletal muscles or liver causes insulin resistance. Studies have shown that rats develop insulin resistance as a result of accumulated lipids in the liver and skeletal muscle followed by lipid/heparin infusions or short-term high-fat diet (HFD) [34]. Furthermore, peripheral insulin resistance and accumulation of lipids were the outcomes of overly expressed lipoprotein lipase (LPL) within the liver or muscles [35,36]. Additionally, LPL deletion within skeletal muscle enhanced insulin signaling in muscles exposed to high-fat diet challenges [37]. Furthermore, in skeletal muscle, insulin-mediated glucose uptake improved by removing proteins that transport fats, such as CD36 or FATP (fatty acid transport protein)-1 [38,39]. Moreover, HFD-induced hepatosteatosis was significantly reduced and glucose tolerance was enhanced by FATP5 or liver-specific knockdown of FATP2 [40,41].

Lipodystrophy is characterized by a significant reduction in fat cells within adipose tissue, leading to ectopic fat deposition in peripheral areas [6]. This condition results in severe insulin resistance and hypertriglyceridemia. These clinical features provide clear evidence that ectopic lipid accumulation plays a role in insulin resistance, independent of any contribution from visceral adipose tissue [6].

Diacylglycerol (DAG)

Numerous studies have observed the impact of abundant accumulation of lipids on insulin signaling in the liver and muscle, concentrating on the downregulation of surface insulin receptors and the damaged transduction of insulin signals in insulin resistance associated with obesity [42]. Insulin resistance in skeletal and liver tissues is thought to be caused by the variety of metabolites of lipids that includes lysophosphatidic acid (LPA), acylcarnitines, ceramides, and diacylglycerol (DAG). This is the most widely accepted theory regarding the ectopic lipid accumulation causing insulin resistance.

Through lipogenesis, fatty acids are promptly esterified inside cells to produce fatty acyl-CoA, which is subsequently carried to a glycerol backbone to produce DAGs, LPA, and triacylglycerols (TAGs). Diacylglycerol (DAG), in particular, is a lipid intermediate that may act as a second messenger in important signaling pathways linked to the emergence of insulin resistance [43]. Rodents that are genetically predisposed to obesity and those subjected to a high-fat diet demonstrated hepatic insulin resistance alongside increased hepatic diacylglycerol (DAG) levels [44,45]. Furthermore, phorbol myristate acetate (PMA), a DAG, decreased the activity of insulin-stimulated glycogen synthase (GYS) and insulin receptor tyrosine kinase (IRTK) in hepatocytes [46].

The DAG theory suggests that DAG buildup in insulin-sensitive tissues leads to lipid-induced insulin resistance, which is caused by protein kinase C (PKC) interfering with insulin signaling. The PKC family comprises three groups: atypical, conventional, and novel. Interestingly, DAG has a much higher affinity for novel PKC (nPKC), which is known to play a part in mediating DAG’s effect on insulin resistance [47,48]. 

Ceramide

Ceramide is classified as a sphingolipid. It is also an important bioactive lipid that is generated by intracellular fatty acids together with sphingosine. It is believed to be implicated in modulating insulin resistance by lipids [49]. Ceramide is essential for regulating the distribution of signaling molecules and maintaining the stability of cell membranes. Hepatic ceramides and homeostatic model assessment for insulin resistance (HOMA-IR) scores are associated with people dealing with obesity [50]. Additionally, elevated hepatic and muscular ceramide levels have been routinely detected in obese rats having insulin resistance [44]. The use of myriocin, a serine palmitoyltransferase inhibitor, effectively inhibited ceramide synthesis, thereby preventing insulin resistance and reducing ceramide levels in mice subjected to a high-fat diet [51,52]. Dihydroceramide desaturase 1 (Des1), a crucial regulator of ceramide production, was heterozygous in mice which exhibited decreased levels of total ceramide in the liver and low fasting HOMA-IR scores [51]. Increased glucose tolerance conferred protection against obesity induced by a high-fat diet, and lower hepatic ceramide levels, especially of the C16:0 species, were the outcomes of liver-specific knockout of ceramide synthase 6 (CerS6) deletion [53]. Additional mechanisms that aid in the comprehension of insulin resistance are endoplasmic reticulum (ER) stress and systemic chronic inflammatory response [54-56].

Genetic variants and the role of pregnane X receptor in NAFLD pathogenesis

Genome-wide association studies (GWAS) have revealed several genetic variants associated with NAFLD, including PNPLA3 and TM6SF2, both playing significant roles across the disease spectrum. The PNPLA3 I148M variant has been strongly linked to increased hepatic fat content, progression to steatohepatitis, and fibrosis. This gene variant affects triglyceride metabolism, leading to hepatic lipid accumulation. Similarly, the TM6SF2 variant (rs58542926) reduces very low-density lipoprotein (VLDL) secretion, resulting in fat retention within the liver and promoting NAFLD progression [57,58].

The pregnane X receptor (PXR) also plays a pivotal role in NAFLD pathophysiology by regulating lipid metabolism and interacting with the gut-liver axis. Activation of PXR exacerbates hepatic steatosis and inflammation, particularly in obesity-induced models. PXR promotes weight gain and modulates the gut microbiome by increasing pro-inflammatory bacteria, such as *Lactobacillus*, and reducing beneficial species like *Bifidobacterium*. Its activation has been linked to increased oxidative stress and inflammation in the liver, positioning PXR as a promising target for therapeutic interventions in NAFLD​ [59].

The interplay between NAFLD, IR, and MetS

The complex interplay between non-alcoholic fatty liver disease (NAFLD) with metabolic syndrome (MetS) as well as insulin resistance (IR) is characterized by a reciprocal relationship, where each disorder aggravates the others, creating a self-perpetuating cycle of metabolic dysregulation. NAFLD is often considered the MetS’s hepatic manifestation, as the two share overlapping pathophysiologies, primarily driven by IR. MetS, defined by a bundle of disorders, including central obesity, glucose intolerance, dyslipidemia, and hypertension, serves as a catalyst in this dynamic, amplifying the progression and severity of NAFLD through multiple metabolic and inflammatory pathways [60,61].

IR is a central pathological feature in both MetS and NAFLD. It disrupts the normal metabolism of glucose and lipids, causing hyperinsulinemia, elevated lipolysis, along with an overproduction of free fatty acids (FFAs) by adipose tissue [59,60]. The liver, overwhelmed by the influx of FFAs, increases de novo lipogenesis while reducing fatty acid oxidation and very low-density lipoprotein (VLDL) export, leading to triglyceride accumulation within hepatocytes. This lipid overload induces lipotoxicity, triggering mitochondrial dysfunction, endoplasmic reticulum stress, and oxidative stress, which activate pro-inflammatory passage of signaling like the nuclear factor kappa B (NF-κB) and c-Jun N-terminal kinase (JNK) pathways. These mechanisms sustain fibrosis and hepatic inflammation, hastening the progression of fibrosis and the transition from simple steatosis towards non-alcoholic steatohepatitis (NASH) [62,63].

MetS further exacerbates NAFLD through its association with visceral adiposity and the chronic low-grade inflammation it induces. Tumor necrosis factor-alpha (TNF-α) and interleukin-6 (IL-6) are two pro-inflammatory cytokines and adipokines released by visceral fat, which elevates hepatic inflammation and systemic IR [61,62]. Dysregulated adipokines, such as adiponectin and leptin, impair insulin sensitivity and lipid metabolism, worsening hepatic fat accumulation and inflammation. Moreover, hypertrophied adipocytes in MetS release monocyte chemoattractant protein-1 (MCP-1) that enrolls macrophages toward adipose tissue and the liver, perpetuating inflammatory cascades [64,65].

The liver does not function in isolation; its interplay with other organs, particularly adipose tissue along with the gut, is crucial during the pathogenesis of NAFLD. Gut dysbiosis, characterized by an imbalance in the gut microbiota, causes lipopolysaccharides (LPS) to move into the portal circulation and promote intestinal permeability. LPS activates hepatic toll-like receptor (TLR) pathways, promoting inflammation and IR. This gut-liver axis dysfunction is further implicated in the worsening of NAFLD in the context of MetS [66,67].

Self-perpetuating metabolic feedback loops underlie the chronicity of NAFLD. IR promotes the release of FFAs and inflammatory cytokines, which further impair insulin signaling and exacerbate hepatic steatosis and inflammation. Oxidative stress generated during lipotoxicity enhances the synthesis of reactive oxygen species (ROS), amplifying liver injury and fibrosis. These pathological interactions, compounded by the metabolic disturbances of MetS, establish a vicious cycle that perpetuates and intensifies NAFLD progression [68].

Understanding the bidirectional and synergistic relationships among NAFLD, IR, and MetS provides insights into potential therapeutic strategies. Targeting IR, modulating adipokines, restoring gut microbiota balance, and mitigating inflammation are promising approaches to breaking this pathological cycle, offering hope for effective management of NAFLD and its systemic metabolic consequences. 

Clinical implications

Early detection and treatment of NAFLD is important to prevent progression to cirrhosis. At present, cirrhosis is rated as the 11th most common mortality cause globally [69]. The identification of biochemical and molecular biomarkers implicated in the advancement from non-alcoholic fatty liver (NAFL) towards non-alcoholic steatohepatitis (NASH) is essential for reducing mortality rates among NASH patients with fibrosis. Cirrhosis and death from cardiovascular or liver-related causes are more likely to occur in patients having NASH [70]. It is possible to prevent the progression from NAFLD to cirrhosis and its associated complications by determining the stage of hepatic fibrosis [70]. Common biomarkers in clinical practice fall into three categories which are (a) imaging biomarkers for fibrosis, (b) indirect biomarkers of hepatic fibrosis, and (c) direct biomarkers of hepatic fibrosis [70].

The primary identification of non-alcoholic fatty liver disease (NAFLD) is acquired from laboratory results, particularly elevated aminotransferase and gamma-glutamyl transferase levels. Additional imaging studies are required after ruling out secondary causes of fatty liver, such as autoimmunity, viruses, drugs, and alcohol.

An ultrasound-based method called transient elastography (TE) is used to screen NAFLD in patients who have more than one metabolic syndrome (MetS) constituent. The findings of TE data are shown on screen as liver stiffness measurement (LSM) and controlled attenuation parameter (CAP) [71]. Several researches have demonstrated that the presence of arterial hypertension, T2DM, waist circumference, BMI, and the number of MetS components all significantly raise CAP measures [68]. Patients should be referred to diabetologists, hepatologists as well as nephrologists for consultation once TE with CAP is used to diagnose NAFLD [71].

Lifestyle changes, pharmacotherapy, and surgical interventions are among the treatment options for NAFLD and obesity. Nonetheless, the only effective treatment for NAFLD currently is weight loss. Reduction of dyslipidemia and inflammation, weight loss, and normalization of insulin levels are among the benefits of However, whether bariatric surgery directly contributes to the treatment of NAFLD or not is still uncertain [72]. 

Challenges and future directions

The complex interplay among non-alcoholic fatty liver disease (NAFLD) with metabolic syndrome (MetS) as well as insulin resistance (IR), presents several challenges that hinder effective treatment and prevention. One key challenge is the lack of knowledge on the precise mechanisms that propel NAFLD’s advancement from simple steatosis to more complex forms like cirrhosis and non-alcoholic steatohepatitis (NASH). While IR is recognized as a major contributor to NAFLD development, the multifactorial nature of MetS complicates the identification of specific pathways that could be targeted therapeutically [66]. Additionally, the variability in patient response to lifestyle interventions and pharmacological treatments highlights the need for more individualized therapeutic approaches [64].

Future research should focus on identifying novel biomarkers that can help monitor NAFLD and its early detection, especially in its silent stages. The development of non-invasive diagnostic tools, such as advanced imaging techniques or serum biomarkers, could reduce reliance on liver biopsies [61]. Furthermore, research into targeted therapies that address specific metabolic pathways involved in IR and NAFLD is crucial. Understanding the molecular mechanisms linking gut microbiota, adipose tissue, and the liver could open new avenues for therapeutic interventions aimed at disrupting the self-perpetuating cycles of inflammation and fat accumulation [67].

## Conclusions

In conclusion, this review highlights the strong interconnection between non-alcoholic fatty liver disease (NAFLD) and metabolic syndrome (MetS) as well as insulin resistance (IR), illustrating how these conditions contribute to the worsening of liver function and overall metabolic health. NAFLD, often regarded as MetS’s hepatic manifestation, is driven largely by IR, which exacerbates accumulated fat, oxidative stress, and inflammation inside the liver. This cycle can progress NAFLD from simple steatosis to more complex disorders like cirrhosis and non-alcoholic steatohepatitis (NASH). The reciprocal association between MetS and NAFLD emphasizes the need for comprehensive approaches to management, including lifestyle modifications and pharmacological interventions. While there has been advancement in better understanding the basic process, there are still gaps in knowledge, particularly regarding early detection and effective treatment strategies. Future studies should prioritize closing these gaps to efficiently address the prevailing NAFLD and its metabolic complications globally.
